# Whether Social Participation Can Affect the Central Government Public Policy Response to the COVID-19 in China

**DOI:** 10.3389/fpubh.2022.842373

**Published:** 2022-04-27

**Authors:** Liping Fu, Huajun Sun, Kaibo Xu

**Affiliations:** ^1^College of Management and Economics, Center for Social Science Survey and Data Tianjin University, Tianjin, China; ^2^College of Politics and Public Administration, Qinghai Minzu University, Xining, China; ^3^School of Public Policy and Management, Tsinghua University, Beijing, China

**Keywords:** public participation, government response, COVID-19 pandemic, governance, media

## Abstract

**Objective:**

The coronavirus disease 2019 (COVID-19) outbreak has threatened the state's governance and public safety. This study investigates whether and how public participation can affect central government policy response to this pandemic in the context of China.

**Methods:**

This study constructs the theoretical framework based on theoretical analysis, and Social Network Analysis is used to analyze data on the public participation, New Media, and the central government response in this pandemic.

**Results:**

Findings show that the Chinese central government does not always adopt top-down elitist governance strategies in risk management. The central government will also adopt the bottom-up governance strategy triggered by public participation. In this process, New Media acts as a catalyst. Specifically, when faced with a public emergency and needs a policy response from the central government, public participation firstly creates “participation” with the authority of the media, forms public opinion, and then the prompt policy response from the central government.

**Conclusion:**

This study confirms that the central government will refer to public participation to decide the policy response. It also shows that the theory of government response applies to both the local government and the central government.

## Introduction

Coronavirus disease 2019 (COVID-19) has been declared a global pandemic (1). The pandemic has spread to 213 nations, devastating impacting economies and health systems (2). COVID-19 was discovered and first reported in China in December 2019 in Wuhan (3). The pandemic is very infectious and may be spread via the respiratory tract (4). It is also extremely contagious and will be spread through close contact. COVID-19, as a characteristic of high-risk management, presents a challenge for governments in terms of fast and correct administrative measures and risk communication management. Faced with the pandemic, all countries adopt different strategies to deal with this risk management, especially China. As one severely affected countries, China takes large-scale public health interventions, including lockdown and home isolation (4). As we all know, when huge catastrophes or emergencies strike, top-down governance strategies are frequently employed, with the central government's response always setting the precedent in China. However, with the increasing complexity of social problems, this strategy may face many challenges. Bottom-up governance initiatives are gaining attraction. Bottom-up governance strategy triggered by public participation has become a trend in the western developed countries, particularly during times of public emergency, as governments pay growing heed to the democratic will, particularly during times of public emergency. Indeed, public participation in local government administration has been critical in China. Public participation in central government governance, on the other hand, has received little attention. As a result, this study tries to investigate whether the central government will also adopt a bottom-up governance strategy triggered by public participation in an emergency. Previous research expressed similar opinions but did not provide a concrete explanation; this makes it difficult to reach a conclusive conclusion.

To this end, this study seeks to investigate whether and how public participation can affect the central government's policy response during a Chinese emergency. This study begins by developing a novel theoretical framework for how public participation influences the central government's governance behavior in the setting of central-local interaction; this study then discusses the case of COVID-19. Within this context, this study examines how public participation influences the policy response of the central government. When the public participation attempts to influence the “upper” central governments, they generate “participation” to create a social effect and then enlist the media's authority to shape public opinion, thereby triggering the central government's behavior to respond to the local governments. As a result, this study suggests an alternative to central control that incorporates public engagement in times of emergency or pandemic.

## Literature Review And Theoretical Framework

### Government Response to Public Participation

Public participation and government response are two critical aspects of democratic processes. A central claim of democratic theory is that democracy induces governments to respond to people's preferences (5). Liberal democracies have faced significant challenges over the last few decades, compelling scholars to generate fresh insights into the democratic connection; as a result, there is a growing body of study on the relationship between public participation and government governance. Additionally, an increasing number of scholars have established that regimes strategically employ government response to the public as part of their efforts to retain power (6). People express their opinions on topics and expect governments to address their concerns through public policy; hence, viewing opinion polls as a tool for public action can open new possibilities. Public participation is a means of ensuring that citizens have a direct voice in public decisions. The terms “citizen participation” and “public participation” are often used interchangeably. While both are generally used to indicate a process through which the public has a voice in public policy decisions.

The roots of public participation can be traced to ancient Greece and colonial New England. Prior to the 1960s, governmental processes and procedures were designed to facilitate “external” participation. Public participation was institutionalized in the mid-1960s by President Lyndon Johnson's Great Society programs. A government is considered responsive if “it adopts policies that are signaled as preferred by citizens through public opinion, campaigns, or other ways” (7). Government response literature emerged in American politics and later spread to European politics (8–11). Subsequently, the number of studies on this subject has increased, and numerous papers have expressed similar views, which can influence how governments implement their policies. Simultaneously, governments view public opinion as a critical democratic tool that may supplement a country's existing democratic structure.

Public participation and government response are not exclusive to American politics and western countries. Asia has also increasingly experimented with limited forms of public participation in recent years, resulting in a range of “hybrid” regimes. This has effectively shown governments' public participation and response in state systems. China is crucial because its government is increasingly saturated with a diverse array of participatory and deliberative activities (12–14). China has begun debating how the government should respond. Chinese politics has also taken a more deliberate turn regarding how the government responds to public demands and wishes in recent years. This is critical in raising protesters' worries about the policymaking agenda (14, 15). A critical elite-mass linkage in Chinese political affairs is that authorities are more likely to agree to their subordinates' demands when extensive pressure is applied. However, public participation and government response received little emphasis in the beginning. Since then, the reality that technology is progressively influencing public participation has been recognized, even if technological advancements have not bridged the public and government divide. Numerous governments prefer to exclude or minimize public participation in planning initiatives because it is too costly and time-consuming. However, as technology advances, the issue of public participation is gradually being resolved. One critical action in this governance response process is information collection. The New Media is critical in this process because information obtained through mass media becomes common knowledge, making it easier to mount a concerted challenge; in light of these considerations, an regime needs to manage the media to maintain stability (16–19). The development of New Media has enabled the media to collaborate, form a unified force for public opinion guidance, and reshape the expression space for public participation in governance; this can compensate for the institutional lack of public participation in “upper” governance (20).

### Theoretical Framework and Hypotheses

**Figure 1:**
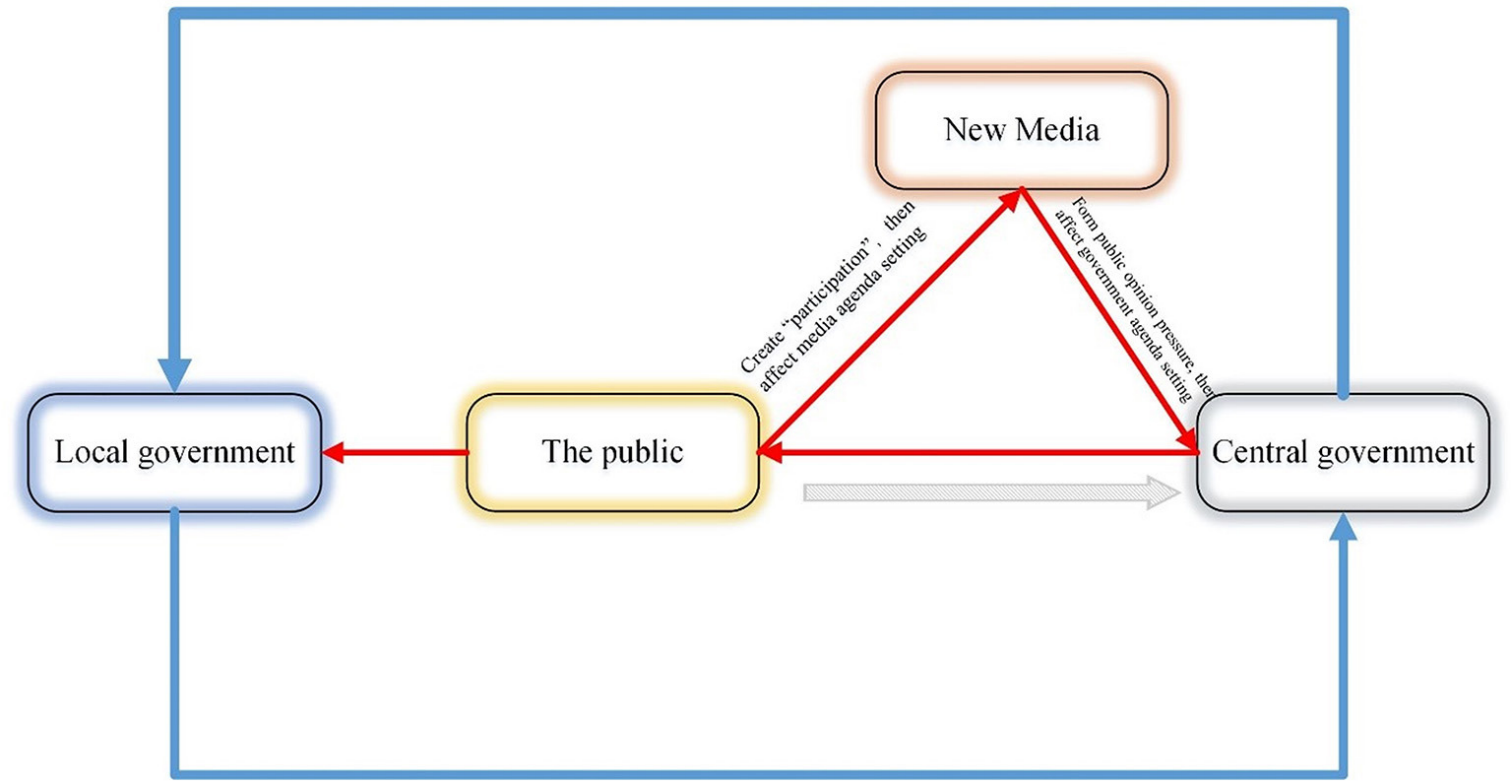
The theoretical framework of public participation in central government response.

Based on government response theory and the evolution of media technology. This study examines how the public attempts to influence the central government's governance behavior. The theoretical framework is depicted in Figure 1; blue represents a top-down governance strategy triggered by central governance; red represents a bottom-up governance strategy triggered by public participation. Specifically, in this red one, the public first affects the media agenda by creating “participation.” Furthermore, the release of information by New Media can create responsive pressure on the central government. The central government responded by generating governance behavior. To explain the theoretical framework, this study takes the COVID-19 pandemic as a case study to demonstrate the public participation's effect on the central government in the context of the central-local relationship. Specifically, to explain social participation in the central government's response, this study constructs three sets of data (Public participation, New Media attention, government response) to indicate the degree of attention paid to this pandemic. For research purposes, this study proposes that the peak of public participation was presumed to occur earlier than the peak of New Media attention to indicate that the media's attention in the pandemic may be affected by public participation. Thus, we propose the following hypothesis.

**Hypothesis 1**: The peak of public participation is slightly earlier than the peak of New Media attention.

Like the hypothesis above, we presumed that the peak of New Media attention occurred before the turning events of the central government's response to indicate that the central government response is under the pressure of media attention. Thus, we propose the following hypothesis.

**Hypothesis 2**: The peak of New Media attention occurs slightly earlier than the turning point of the central government's response.

## Methods And Data

### Case Study and Social Network Analysis

This study takes COVID-19 to present the theoretical framework (how public participation affects central government response). It focuses on how the public influences the central government's response in the context of central-local relations in China. The COVID-19 pandemic was chosen as the case for the following reasons; First, the COVID-19 pandemic is a public health emergency incident that first broke out in Wuhan, Hubei. As a local public emergency, territorial management should have been prioritized. However, such a sudden and complex public emergency is beyond the power and responsibility of the local government and requires support from the central government. In such an emergency, where the occurrence environment is highly complex, the central government often considers the interests of the whole of society before triggering the decision-making system. Given this context, this study explored whether and how public participation affects the central government response. Second, the COVID-19 pandemic is an emergency public event with a substantial spreading effect. The trend is likely to occur in a short period, making the central government's response and public participation in the event more visible. Third, in this pandemic, most citizens were alone at home, with few opportunities for public participation. The majority of those interested in COVID-19 do so through internet searches and social donations. Public participation statistics are readily available and have a high accuracy, which is also a significant reason this study chose this case.

In addition, this study uses social network analysis to analyze the central government's response in these three stages. The main feature of this method is the analysis of the relationships among participants in an event (21, 22). We use this method to explain the specific behaviors of the central government in different stages of the COVID-19 pandemic. These data were used to describe the central government's response intensity. Network structure indexes, such as network nodes, network density, network centrality, and agglomeration subgroups, represented the central government's response to the pandemic.

### Data

The equations should be inserted in editable format from the equation editor. According to the theoretical framework proposed in this study, we need to construct three indicators. Data represent the central government's response, public participation, and New Media attention. In this pandemic, local governments initiated the response to the COVID-19 pandemic that required the central government to respond. Therefore, the data collected in this research mainly focused on the central government's response. Specifically, this study primarily collected data on the prevention and control measures of the COVID-19 pandemic that the central government issued. Whenever the central government took relevant pandemic prevention and control measures in Wuhan or Hubei, it was recorded as a central government response. This study defines central government as central state administrative organs and their subordinate institutions. Specific information was then extracted from these critical events, including the event, theme, and time. To ensure the accuracy and comprehensiveness of the obtained data, my collaborators and I searched separately and then compared and screened the data to ensure validity. After collating the data, the chosen study period was from December 31, 2019, to February 15, 2020. We collected 64 critical events related to the central government's response in this period. We invited experts to make judgments to distinguish turning events within this period. Based on experts' opinions, this study selected three key turning events as the starting points of these three stages. These three turning events were also considered the “peaks” of the central government's response, as shown in Table 1.

**Table 1:** Important events related to the Central Government's response in the COVID-19 pandemic.

**#**	**Stage**	**Event**	**Time**	**Subject of Action**
**1**	Stage of Incubation	The expert group of the National Health Commission arrived in Wuhan and launched a formal intervention investigation	12/31/2019	National Health Commission
**2**		The National Health Commission established an outbreak response team	01/01/2020	National Health Commission, Chinese Academy of Medical Sciences, Chinese Academy of Sciences, Academy of Military Sciences
**3**		The National Center for Disease Control and Prevention initiated the first-level response	01/06/2020	National Center for Disease Control
**4**		Xi Jinping put forward requirements for pandemic prevention and control work	01/07/2020	General Secretary
**……**		……	……	……
**16**	Stage of outbreak	Xi Jinping announced important instructions	01/20/2020	General Secretary
**17**		The COVID-19 pandemic was listed under the infectious disease and Health Quarantine Law	01/20/2020	The State Council
**18**		Zhong Nanshan was appointed team leader of scientific research on the pandemic emergency in China	01/21/2020	Ministry of Science and Technology, National Health Commission, National Development and Reform Commission, Ministry of Education et al.
**19**		The National Health Commission advised people not to travel to Wuhan and advised residents of Wuhan not to leave	01/22/2020	National Health Commission
**……**		……	……	……
**54**	Stage of duration	The first joint prevention and control conference of the State Council	02/05/2020	National Health Commission, Ministry of Propaganda, National Development and Reform Commission, Ministry of Industry and Information Technology
**55**		Hesheng Wang was appointed as the Standing Committee of Hubei Provincial Committee	02/08/2020	Department of Human Resources and Social Security
**……**		……	……	……
64		Notice of the Central Committee of the Communist Party of China on the appointment of Yong Ying and Chaoliang Jiang	02/13/2020	Department of Human Resources and Social Security

The data collection on social participation in the COVID-19 pandemic consisted of two parts. One represents the public's participation; In this study, we used the Baidu index of the COVID-19 pandemic daily searches (23) and daily donations of the Hubei province Red Cross (http://www.hbsredcross.org.cn/) to represent public participation. The study included these two indicators because, at that time, due to home isolation, the people focused on the pandemic primarily through Internet searches and donations to Hubei Province. The daily donations were calculated by dividing the total amount donated each day by the number of donations that day. Then, the two types of data were processed in a unified dimension, and the data were compressed within the range of 0,1 by adopting the normalization method (NMS). The results can be presented by two factors, namely the search index and donation index. The calculation formula is as follows:


(1) $$\begin{array}{r} \text{Y}=\left(\text{X}-\text{Min}\right)/\left(\text{Max}-\text{Min}\right) \end{array}$$

The other represents the media's attention. The data of New Media attention is from the media index of the Baidu Index, which means the number of COVID-19 pandemic reports collected in Baidu News every day (23). After the same unified dimensional processing, the data obtained were defined as the media index in this study.

## Results

### Descriptive Statistics

As shown in Table 1, the first stage (December 31, 2019–January 19, 2020) was defined as the incubation period of the COVID-19 pandemic. The first response of the central government was marked by the arrival of the National Health Commission's expert team in Wuhan on December 31, which also marked the beginning of the official intervention. Fifteen critical events related to the central government's response were collected during this first period. The second stage (January 20, 2020–February 4, 2020) was defined as the outbreak stage of this pandemic, which began when the State Council held its first meeting on January 20 in response to the pandemic. This conference was also marked when the COVID-19 pandemic began to receive significant attention from the central government. The third and final stage (February 5, 2020–February 15, 2020) was defined as the stage of the duration, which began with the State Council's first joint prevention and control work on February 5, 2020 (this indicated that various departments of the central government had started to attach great importance to internal cooperation in response to the COVID-19 pandemic and the prevention and control work began to operate regularly). Eleven critical events concerning the central government's response were collected during this stage.

**Figure 2:**
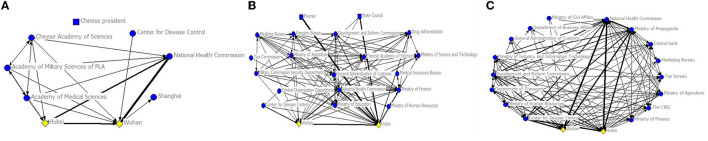
**(A–C)** Response of the central government in three stages.

Figure 2 shows the central government's response throughout these three stages. In this figure, “nodes” represent actors involved in managing the COVID-19 pandemic. These actors include the central government and its functional departments. The links between actors in Figure 2 represent the practical cooperation between different entities when combating the pandemic. As can be seen from the network structure in Figure 2a, the central departments of the Ministry of Health, the Centers for Disease Control, and related scientific research institutions formed network links with Wuhan during the incubation period of the pandemic. At this stage, the central government's response to local governments was not yet apparent. Most known measures involved the central government sending departments to conduct investigations, obtain information, and give instructions without necessary response actions. The network scale was relatively small, with a density of only 0.3472, and only a few central departments provided a critical response.

The network level in Figure 2a was high, with a density of up to 0.6250, indicating an obvious hierarchy among cooperative departments, and the network efficiency was only 0.5238. At this stage, we can conclude that the efficiency of cooperation among various departments was low, and the degree of correlation was also relatively common. However, in the second stage, as shown in Figure 2b, under the leadership of the State Council and other central departments, multi-sectoral cooperation was formed, and regular prevention and control measures were taken. At this stage, the number of network nodes reached 21, and the intensity of the network relationship reached 174. The network density increased significantly compared to the previous step, reaching 0.4143. The power of the network level was reduced considerably, highlighting the cooperation between various central departments. In addition, the network efficiency was higher than that of the incubation period. The intensity of the network level in the outbreak stage is up to 0.5632, which means that the speed of information dissemination between different departments had accelerated, and departments within the network could form associations quickly. At this stage, the central government's response to Hubei and Wuhan mainly focused on specific safeguard measures, including medical supplies, funds, human resources, and specific control measures for Wuhan and Hubei. As shown in Figure 2c, the third stage was the duration of the COVID-19 pandemic when a relatively orderly cooperation mechanism was formed. The joint prevention and control meeting, the number of clinically confirmed cases in Hubei Province, and the data of confirmed cases were included in the daily work schedule. Therefore, the newly formed central government response was significantly reduced at this stage compared to the previous stage, and this trend appeared more stable. The most prominent feature of this stage is that the network efficiency is the lowest among the three stages. With the increasing frequency of central government response, the efficiency of the central government response also decreased. This also confirmed that the efficiency of cooperation declines when frequency increases. In addition to daily work, the main content of the central government response at this stage is reflected explicitly in the adjustment of the core leadership position in Hubei and Wuhan. A linear function method is used to process the data in a unified dimension based on data collation. The donation index, search index, and media index are thereby obtained. The processing results are presented in **Table 3**.

### Mechanism of Public Participation in the Central Government Response

Based on the above theoretical framework, descriptive statistics, and the chronological sequence of the three activities described above, this section will explain the mechanism among public participation, New Media attention and the central government response. The mechanism is defined as follows.

**Figure 3:**
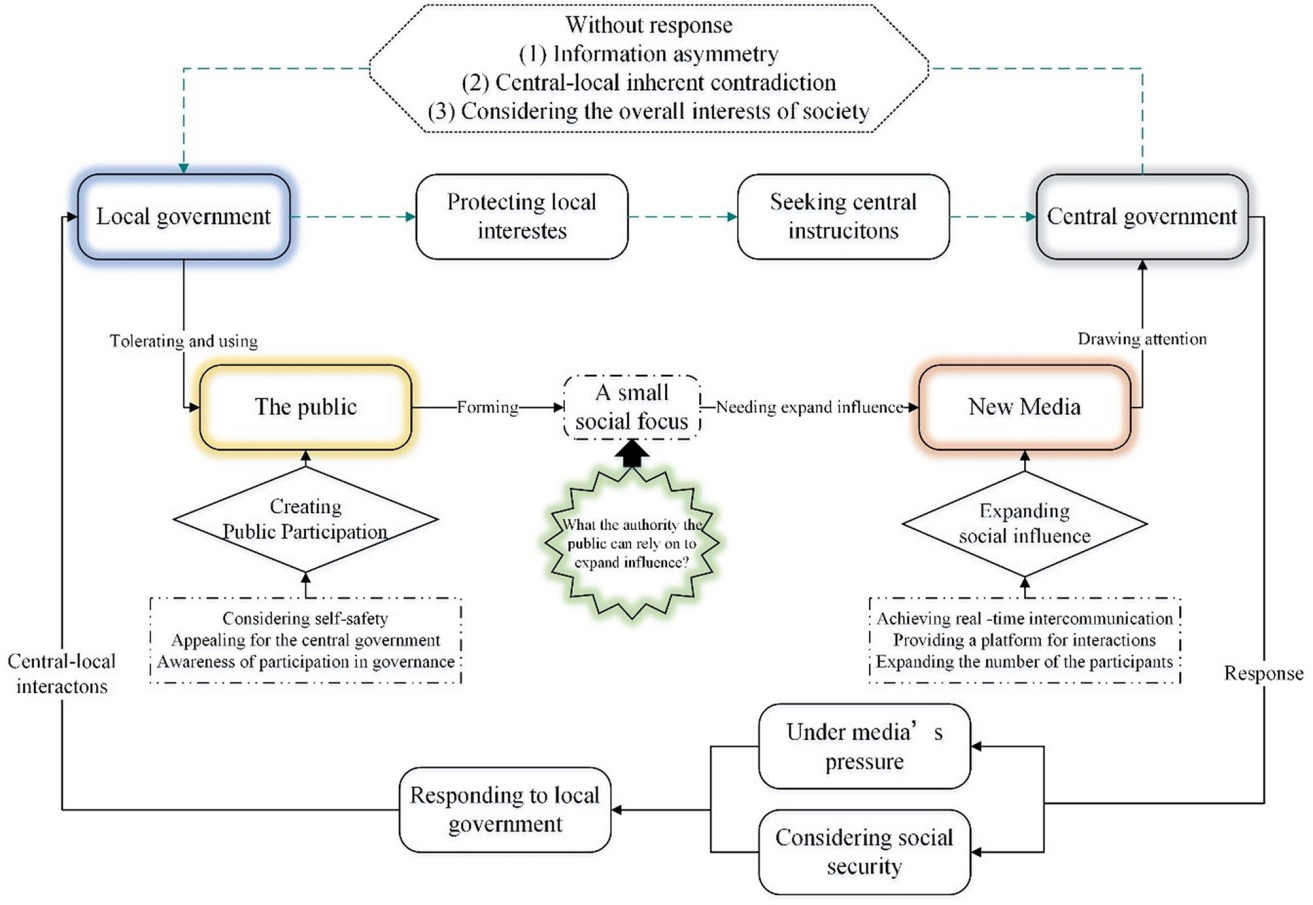
Mechanism of public participation, the attention of new media and the response of the central government in central-local interactions.

Regarding local government works in this mechanism. As is described in Figure 3, in this pandemic, the local governments' power is compressed because of the highly centralized central government in an emergency public health (24); it has not enough ability to deal with this matter. To protect their interests, the local government will seek instructions from the central government to get the freedom of action and support. However, the central government tends to consider the overall interests of the society, the game information asymmetry (25, 26) and central-local inherent contradiction (27); it won't respond right away. This also means that the central-local interactions initiated by the local government failed.

Regarding the public and New Media work in this mechanism. When the local government appeals to the central government but gets no response, the public, as the governance stakeholder, will replace the local government and play a prominent role in the central government response. In a public health emergency, the public will actively participate in social governance based on consideration of self-safety and awareness of participation in governance, appealing to the central government for instructions, even though they have not yet been given formal power and channels to participate (28). Attracting the central government's attention is often challenging because of the lack of legal authority involved in the emergency. They create public participation by making large donations, and following this pandemic, it forms a small social focus. However, we need to find an authority that the public can rely on to expand influence. With the development of technology, the New Media has got significant growth. Its timeliness and quick transmission can make issues in a small scope into social events, provide a platform for interactions, and make everyone a media terminal. So New Media has become an essential tool for the public to participate in governance (29). Through the New media, the public can form public opinion, expand the influence of this pandemic, and attract the attention of the central government.

Regarding the central government works in this mechanism. In the Chinese political system, the decentralization of power between the central government and local governments is principal-agent (30). Especially in emergency public health, the central control is highly centralized. The local authority is limited, so the central government's instruction plays a vital role in this mechanism. However, in such emergency public health, the central government often fails to make immediate decisions considering the overall interests of the society, information asymmetry and other reasons (31). As the representatives of social goods, the public and the media can break the information asymmetry and form public opinion pressure and attract attention from the central government. Under the media's pressure and social security, the central government will respond to local governments and complete the central-local interactions.

### Findings

**Figure 4:**
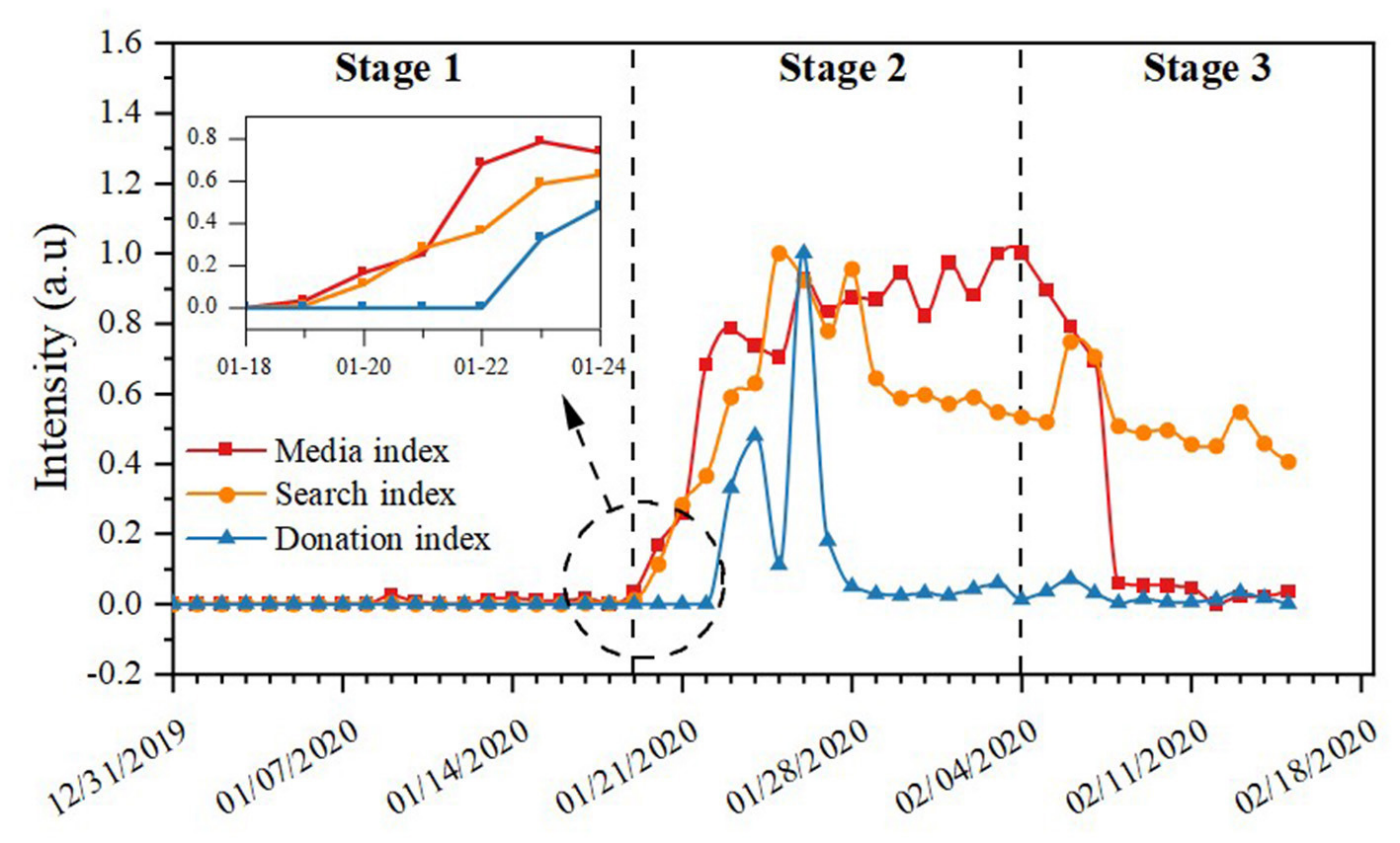
Variations of media index, search index, and donation index in these three stages.

Based on the analysis above, there are two main findings. The central government's response, public participation, and New Media attention are the same as the whole. As shown in **Table 3** and Figure 4^1^, the average and maximum values of the search index, donation index, and media index were lowest during the first phase. As is shown in the first stage of Table 2, regardless of the network scale, the number of networks, or density of the network, the central government's responses were significantly lower than the other two stages, showing that the intensity of the reaction at this stage was also at its weakest compared to different stages. In the second stage, it can be seen from Table 2 that network scale, network relationship number, network density, network efficiency, and network relevance were all at their highest. This indicates that the central government paid great attention to the development trend of the pandemic at this stage and introduced many prevention and control measures.

**Table 2:** Integrated network analysis of the central-local interactions.

	**Network Size**	**Number of Network Relationships**	**Network** **Density**	**Network** **Level**	**Network Efficiency**	**Correlation Degree**
Stage of incubation	9	25	0.3472	0.6250	0.5238	0.7778
Stage of outbreak	21	174	0.4143	0.3333	0.5632	1.0000
Stage of duration	18	164	0.5359	0.2105	0.4044	1.0000

In contrast, the search index, donation index, and media index at this stage presented a linear upward trend around January 20, 2020, indicating that the COVID-19 pandemic received much attention from the public and New Media during this time. In general, regardless of the frequency or degree of public participation, the participation of the media was maintained at a relatively high level. From February 5, 2020, the third stage of COVID-19 began. From Table 3 and Figure 4 above, we can conclude that the intensity of the central government's response at this stage was lower than in the previous stage, and the search index, donation index, and media index also show a significant downward trend. By comparing the changes of the central government's response, and the media index, search index, and donation index in these three stages, the same development trend was maintained, suggesting that the central government's response was synchronous with public and New Media attention in the COVID-19 pandemic. This also shows the relatively complete level of responsive governance in China and the improvement of the ability of democratic construction, which is consistent with the theoretical development of the Chinese government's response.

**Table 3:** Donation index, search index, and media index in three stages.

	**Donation Index**			**Search Index**			**Media Index**		
**Type of data**	**Average**	**Maximum**	**Standard deviation**	**Average**	**Maximum**	**Standard deviation**	**Average**	**Maximum**	**Standard** **deviation**
Stage of incubation	0.0000	0.0000	0.0000	0.0012	0.0027	0.0027	0.0068	0.0370	0.0102
Stage of outbreak	0.1483	1.0000	0.2636	0.6069	1.0000	0.2345	0.7781	1.0000	0.2418
Stage of duration	0.0206	0.0711	0.0210	0.5258	0.7477	0.1067	0.2424	0.8949	0.0210

Second, as proposed in this study's hypothesis, public participation peaked before the peak of New Media attention. New Media attention peaked earlier than the central government's response. In this case, the “peak” of the central government's response, the “peak” of public attention, and the “peak” of New Media attention was the focus of this study. As shown in Figure 4, the public and the media paid attention to the pandemic at a relatively low level during the incubation stage. The intensity of the central government's response at this stage was also standard, and it does not clearly show the relationship between the central government's response and society's attention. There is no prominent “peak,” as shown in the inset image in Figure 4. In the second stage, it can be seen from Figure 4 that the search index, media index, and donation index were all at a high level. As the search and donation indices show, the search and donation index both occurred earlier than the media index until the “peak” on January 28, 2020. After this, the media index showed an opposite trend compared to the search index and donation index.

Furthermore, the media index began to show an upward trend and reached a peak in the second half of this stage. The media index's change is that the public was paying significant attention to the COVID-19 pandemic, so social attention peaked in the first half of the second stage. In the second half of the second stage, the media index peaked. This shows that after experiencing the peak of public participation, great attention was paid to the pandemic on social media, thus forming a peak of New Media attention. This proves hypothesis 1: the peak of public participation occurs earlier than the peak of New Media attention, which indicates that public participation may affect media attention. It can be seen from Figure 4 that the peak of the media index was formed on February 3, 2020, showing that significant attention was being paid to the pandemic on social media. On February 5, 2020, the State Council's first joint prevention and control work conference was launched. This is another crucial “turning” point in the central government's response to the pandemic. We can regard this phenomenon as a government response to media pressure, proving Hypothesis 2. In other words, this result shows that the peak of New Media attention appears before the “peak” of the central government's response. When the central government responded to the policy agenda, the search index, donation index, and media index declined. This phenomenon shows that when the government responds, the public and the media begin to pay less attention to the pandemic, possibly because they recognize its response.

## Discussion

The COVID-19 pandemic in China demonstrates that public participation is critical during a public health emergency. The data reported in the preceding section demonstrate that public participation can impact the central government's policy agenda in COVID-19. In particular, as far as the arrangement structure is concerned, the method by which public participation can affect the policy agenda of the central government at a higher level primarily benefits from social media's expanding effect.

As for the central government's response to public participation, results show that public participation can influence the policy response of the central government, which is consistent with the government response theory. However, this one does not include an *ad hoc* analysis of multiple levels of government. COVID-19 is purposely used in this study to create an environment favorable to high-level government responsiveness to public participation. The findings indicate that public participation can affect the central level. This is referred regarded as the “external pressure” or “quasi-public policy” method of shaping the policy agenda (32). In this approach, characterized by a lack of elitism among participants, a more transparent decision-making process, and limited decision-making influence, the public, the media, and non-governmental organizations (NGOs) all support the government's policy objective (33). However, in terms of decision-making influence, this study's conclusion differs from previous research, indicating the critical role of public participation. According to prior research, public participation is usually informal and devoid of power-sharing; public participation lacks regular and effective avenues for influencing government policies (34). One possible explanation for this outcome is that COVID-19 lacks expert tactics as a newly emerging pandemic. As a result, it received broad attention in a relatively short period. The general populace was able to speak for the elites and influence the central government's policy response.

As for the mechanism of the central government's response to public participation, we learn that social media plays a vital role in this high-level central government response, indicating that technology has accelerated this response process. With the advancement of technology, the media, especially the New Media has become an increasingly powerful tool for monitoring and disciplining state agents in China (35). According to some experts, conducting less research allows for more participatory strategies that foster public participation and interagency collaboration. Thus, additional effort is required to fully use social media's potential for risk reduction promotion (36). Although political elites identify media as a tool for rulers to gather information (37, 38), in this COVID-19 pandemic, a formal channel for public participation is still missing. The authority of public participation forms is used in conjunction with new media to garner the attention of the central government. Thus, if the state has not formally enabled citizens to participate in the national government, this would need the state's emphasis on the media.

The above discussion on public participation and government responses is in the early stages of the outbreak in China. The difference is that Western countries adopted relatively flexible policies early. Still, the potential spread of the pandemic made most liberal democracies in the West, such as Australia, Austria, and Canada et al. all adopt some coercive strategy later. However, this gets violently opposed by the public. France and the Netherlands even employed brutal police force against protesters. It has been proven that an earlier lockdown could save many thousands of lives (39, 40). However, those western countries that have followed the democratic will to ease policy throughout the pandemic, such as Switzerland, have caused higher death rates (41). In Switzerland, it maintained voluntary actions guided by public opinion. The media lacks investigative journalism and fails to question or hold the public health agency accountable (42). Chinese strategy was seen as being highly successful and superior to the approach of democracies at an earlier stage (43). We can also conclude that whether the government conforms or goes against the public opinion, coercive strategy is the better choice in the earlier stage.

However, whether complying with or defying public opinion, the countries discussed above suggest that coercive policies are the wise choice in the earlier stage. But scholars propose that a permanent coercive policy effect is up in the air. Kissler et al. predicted the need for intermittent lockdowns occurring 75% of the time, even after July 2022, which is more likely COVID-19 will be an annual occurrence (44). For the long-term effects of COVID-19, success will ultimately depend not only on the pandemic's biomedical evolution, but also on its social, political and economic impact (45–47). Therefore, some scholars propose that the blockade is not a long-term solution and herd immunity appears to be the only exit from the response to COVID-19. This can be achieved naturally or through a vaccine. For the reasons given here, the lockdowns may only delay the inevitable. The economic recession can cause far more loss of life and well-being over the long run than COVID-19 can (43).

Moreover, low-income countries are more vulnerable to lockdown. However, many scholars have also opposed this method of herd immunity. Such herd immunity, which requires an entirely liberal policy, will exacerbate health inequalities in the high-risk groups (48), especially those with limited health resources and the elderly, which increases their mortality. Therefore, Scholars have proposed that based on the long duration of COVID-19, different policies should be adopted for different public opinions to ensure economic growth and protect the vulnerable groups (43). For example, particular protection policies should be implemented for the elderly, and children with low mortality should be allowed to return to school usually. Therefore, governments response should be adjusted according to the stage of the pandemic and the reasonableness of public opinion to improve the efficiency of government response. Therefore, it was wise for China to follow public opinion and take control measures in the early stage. However, the long-term negative impact of pandemic prevention may lead to the opposite outcome. China can follow the strategy discussed above and adjust the current coercive policy.

This result has implications for public participation in government response theory and practice. In theory, this article contributes to the growing knowledge about public participation in governance. Public participation in governance has become a common phenomenon, and State–Society collaboration has become the norm (49). This study confirms previous research that the public's participation can make up for the deficiency of government governance, enhance the effectiveness and response of government governance, and make up for the “policy implementation gap” (50). Especially in public health emergencies, it is essential to develop the ability to work with stakeholders. On practical implications. As discussed above, New Media can bridge the gap between public and central governments as a tool. When the central government is confronted with a public emergency, it is unclear when the response mechanism should be activated (51). The central government can refer to the public and the media to indicate when to respond (52). The theoretical framework proposed in this study is a way for the public to affect “upper” governance. Even if a mature expression platform and mechanism have not been formed, the public can still receive the media's attention by creating public participation. Then the media can shape public opinion to try and get the attention of the central government. As far as the government is concerned, public participation and media attention give essential signals for the central government to make governance decisions in public emergencies. While this study adds to our understanding of public participation in central government, it does have limitations. The article's data on public participation comes from the Baidu Index, which effectively disregards those of the general population who do not utilize the internet. According to the statistics available, their attention has been diverted away from the pandemic.

## Conclusions

Through a theoretical construction and a case analysis of COVID-19, this study investigates whether and how public participation affects the central government's response policy. This study situates it in the context of local governments' ineffectual appeals to the national government and the public's attempt to respond to the central government. Results show that public participation can influence the central government's policy agenda by creating social participation. In this process, the central government's response results from the spread of New Media. This study adds to our understanding of government response theory. This article focuses on the central government's response and demonstrates that public participation can also impact the central government's policy response.

In contrast to prior research on government response, this study focused on breaking down the different levels of government response. It focused on the central government's response to the public. In practice, this study proposes that when the government is confronted with an emergency, the absence of referable experience and specialists may increase the importance of public participation in the government's policy response. This result can serve as a reference for the government's response to public emergencies. However, governments should also selectively respond to the public and make a wise choice in the face of unreasonable demands from the public.

This study also has limitations. Such governments' responses at different levels are only carried out in China's context, and governments' responses at different levels in different countries to public participation need further study. In addition, compared with traditional government governance procedures, future studies need further to evaluate the efficiency of such public participation in governance.

## Data Availability Statement

The datasets presented in this study can be found in online repositories. The names of the repository/repositories and accession number(s) can be found at: http://www.hbsredcross.org.cn.

## Author Contributions

KX and HS: methodology. KX: software. HS: validation, resources, and writing—review and editing. LF, KX, and HS: formal analysis. LF: data curation, writing—original draft preparation, visualization, supervision, project administration, and funding acquisition. All authors have read and agreed to the published version of the manuscript.

## Funding

This research was funded by the National Social Science Foundation of China, grant number 20AGL034.

## Conflict of Interest

The authors declare that the research was conducted in the absence of any commercial or financial relationships that could be construed as a potential conflict of interest.

## Publisher's Note

All claims expressed in this article are solely those of the authors and do not necessarily represent those of their affiliated organizations, or those of the publisher, the editors and the reviewers. Any product that may be evaluated in this article, or claim that may be made by its manufacturer, is not guaranteed or endorsed by the publisher.

## Footnotes

^1^The donation on January 26, 2020, is relatively high because there are a few rather large donations on that day.
